# Inflammation and Infection Do Not Promote Arterial Aging and Cardiovascular Disease Risk Factors among Lean Horticulturalists

**DOI:** 10.1371/journal.pone.0006590

**Published:** 2009-08-11

**Authors:** Michael Gurven, Hillard Kaplan, Jeffrey Winking, Daniel Eid Rodriguez, Sarinnapha Vasunilashorn, Jung Ki Kim, Caleb Finch, Eileen Crimmins

**Affiliations:** 1 Department of Anthropology, University of California Santa Barbara, Santa Barbara, California, United States of America; 2 Department of Anthropology, University of New Mexico, Albuquerque, New Mexico, United States of America; 3 Department of Anthropology, Texas A&M University, College Station, Texas, United States of America; 4 Departamento de Medicina, Universidad Mayor de San Simón, Cochabamba, Bolivia; 5 Andrus Gerontology Center, University of Southern California, Los Angeles, California, United States of America; University of Utah, United States of America

## Abstract

**Background:**

Arterial aging is well characterized in industrial populations, but scantly described in populations with little access to modern medicine. Here we characterize health and aging among the Tsimane, Amazonian forager-horticulturalists with short life expectancy, high infectious loads and inflammation, but low adiposity and robust physical fitness. Inflammation has been implicated in all stages of arterial aging, atherogenesis and hypertension, and so we test whether greater inflammation associates with atherosclerosis and CVD risk. In contrast, moderate to vigorous daily activity, minimal obesity, and low fat intake predict minimal CVD risk among older Tsimane.

**Methods and Findings:**

Peripheral arterial disease (PAD), based on the Ankle-Brachial Index (ABI), and hypertension were measured in Tsimane adults, and compared with rates from industrialized populations. No cases of PAD were found among Tsimane and hypertension was comparatively low (prevalence: 3.5%, 40+; 23%, 70+). Markers of infection and inflammation were much higher among Tsimane than among U.S. adults, whereas HDL was substantially lower. Regression models examine associations of ABI and BP with biomarkers of energy balance and metabolism and of inflammation and infection. Among Tsimane, obesity, blood lipids, and disease history were not significantly associated with ABI. Unlike the Tsimane case, higher cholesterol, C-reactive protein, leukocytes, cigarette smoking and systolic pressure among North Americans are all significantly associated with lower ABI.

**Conclusions:**

Inflammation may not always be a risk factor for arterial degeneration and CVD, but instead may be offset by other factors: healthy metabolism, active lifestyle, favorable body mass, lean diet, low blood lipids and cardiorespiratory health. Other possibilities, including genetic susceptibility and the role of helminth infections, are discussed. The absence of PAD and CVD among Tsimane parallels anecdotal reports from other small-scale subsistence populations and suggests that chronic vascular disease had little impact on adult mortality throughout most of human evolutionary history.

## Introduction

We report the first systematic study of peripheral arterial disease (PAD), hypertension and cardiovascular risk factors in a population with both high infectious and parasitic burden but low adiposity and robust physical fitness. The Tsimane are a population of 9,000 forager-horticulturalists in the Bolivian Amazon. Their recent life expectancy at birth of 43 years resembles much of Europe in the mid-nineteenth century, with half of documented deaths by infectious and parasitic disease [1]. The Tsimane have only in the past decade begun an epidemiological transition to increased life expectancy. Public health infrastructure and medical services remain minimal for most Tsimane and they continue to incur high rates of infection. Immunization was only regularly administered in the past decade and is still sporadic in many areas. Medical surveys show that about one-third suffer from respiratory illness, one-fourth from gastrointestinal illness, and over three-fourths from intestinal helminths or pathogenic protozoa. Anemia is highly prevalent and physical growth is stunted. Their history of infection and chronic low-grade inflammation lead us to predict greater PAD and hypertension.

Prevalence of PAD and hypertension was measured in Tsimane adults and compared with representative samples from seven countries spanning the Americas, Asia, Africa and Europe. PAD was assessed with the Ankle-Brachial Pressure Index (ABI), a simple, non-invasive and recommended form of PAD diagnosis [2] and risk indicator of coronary heart disease, stroke and mortality [3], [4], [5], [6]. We use regression models to examine associations of ABI and blood pressure with cholesterol, triglycerides, and body mass index (BMI) (measures of energy balance) and with biomarkers of inflammation [C-reactive protein (CRP)] and infection [erythrocyte sedimentation rate (ESR), leukocytes (WBC)]. We present comparative analyses among U.S. adults using a large, nationally representative sample (NHANES) in order to highlight the distinctions between Tsimane and members of a sedentary, well-fed and low infection population.

### Background and Significance

Cardiovascular disease (CVD) and stroke account for the majority of adult deaths in the industrialized world, and are now major causes of morbidity and mortality in the developing world [7]. In the U.S., atherosclerosis is the main cause of CVD-related heart attacks and strokes [8]. PAD, affecting limb circulation in 10 million Americans, is primarily caused by atherosclerosis and is a risk indicator for coronary and carotid arterial disease, aneurysm, diabetes, and hypertension [9]. Diagnosis of PAD increases risk of CVD mortality 4–6 times over healthy age-matched individuals, though PAD is often asymptomatic [6]. The prevalence of PAD is 10–25% in men and women over 55 in developed countries.

Recent research considers chronic inflammation in the onset and progression of CVD. Many studies associate inflammatory markers and CVD morbidity and mortality after controlling for risk factors [8], [10], [11]. Bacterial, viral and parasitic infections are common among traditional human and primate populations both now and throughout our evolutionary past [12]. As a consequence, inflammation and adaptive immune responses have evolved under intense selection against pathogens. However, in modern societies with immunizations, public sanitation, adequate nutrition and medical services, and where hypertensive people live sedentary lives and frequently smoke, inflammatory processes have been implicated in all stages of arterial aging and atherogenesis [10], [11], beginning early in life [10], [13], and in the critical CVD risk factor, hypertension [14].

Atherosclerosis and CVD are caused by a complex interaction of lifestyle factors (diet, energy balance, smoking, exercise) and inflammatory pathways. Elevated cholesterol and other risk factors predict less than half of heart attacks annually [15]. The acute phase inflammatory marker, C-reactive protein (CRP), often predicts future vascular events better than other risk factors [16], [17]. Indeed, individuals with lower than average cholesterol but high CRP have fewer heart attacks when given statins [18]. An estimated 25–30 million Americans fall into this category of low cholesterol but high CRP. Although CRP's role either as inflammatory marker or causal agent of vascular disease continues to be a subject of lively debate [19], [20], [21], it is increasingly used in clinical settings to help evaluate CVD risk.

Understanding the interaction of diet, energy balance, physical activity and inflammation is hampered by the populations studied: mainly well-fed in developed nations, or experimental animals fed *ad libidum* on diets that promote rapid growth. Both biomes contain few infectious pathogens and environmental inflammogens. In contrast, past human populations faced environments with strenuous physical demands, greater pathogen burden and low caloric diets. These environments characterize the lives of extant indigenous peoples with traditional lifestyles. Much of total mortality in those environments is by infections and parasites, rather than by the causes common in industrial populations, like chronic heart disease, diabetes and cancer [15], [22]. Thus, opposing risk conditions characterize such populations. On one hand, high infection rates over the life course should associate with greater lifetime inflammation exposure, and hence greater risk of atherosclerosis and CVD. On the other hand, high work effort, minimal obesity, and low fat intake should lower CVD risk.

Though arterial aging in the form of elastin fragmentation and medial fibrosis may be an inevitable outcome of aging, the role of atherosclerosis in adult morbidity before the 20^th^ century remains unclear. Studying arterial disease and CVD in indigenous populations is illuminating for three reasons. First, new light is shed on the roles and interactions of diet, exercise and inflammation on disease etiology. Second, subsistence populations with minimal medical access are characteristic of our evolutionary past, and may be informative about the role of vascular disease in the biology of aging during the long course of human evolution. Third, infectious disease is most prevalent in tropical regions of the developing world where the synergistic mix of risk factors and infectious causes are expected to bring a “gigantic epidemic of heart disease” in the coming decades [23].

Existing research provides contradictory findings and lacks important information on relevant variables. Traditional populations often show negligible CVD prior to acculturation to western diets and sedentary lifestyles [22], [24], [25]. Recently, however, Australian Aborigines and Pima Indians have acquired the highest prevalence of obesity and Type 2 diabetes in the world [26]. Higher rates of atherosclerosis, heart murmurs and other cardiac conditions among US soldiers in early 20^th^ century were linked to higher lifetime burden of infectious disease, particularly respiratory infections and rheumatic fever [27]. In one of the few non-western populations assessed for PAD, high prevalence was found among black South Africans [28], consistent with their extensive CVD mortality and morbidity. To date, no studies have measured the prevalence of PAD and other cardiovascular risk factors, such as hypertension, in an environmental context with chronically low energy status and high rates of infection.

## Results

### Prevalence of CVD: PAD and BP

#### Peripheral arterial disease (PAD)

**Figure 1:**
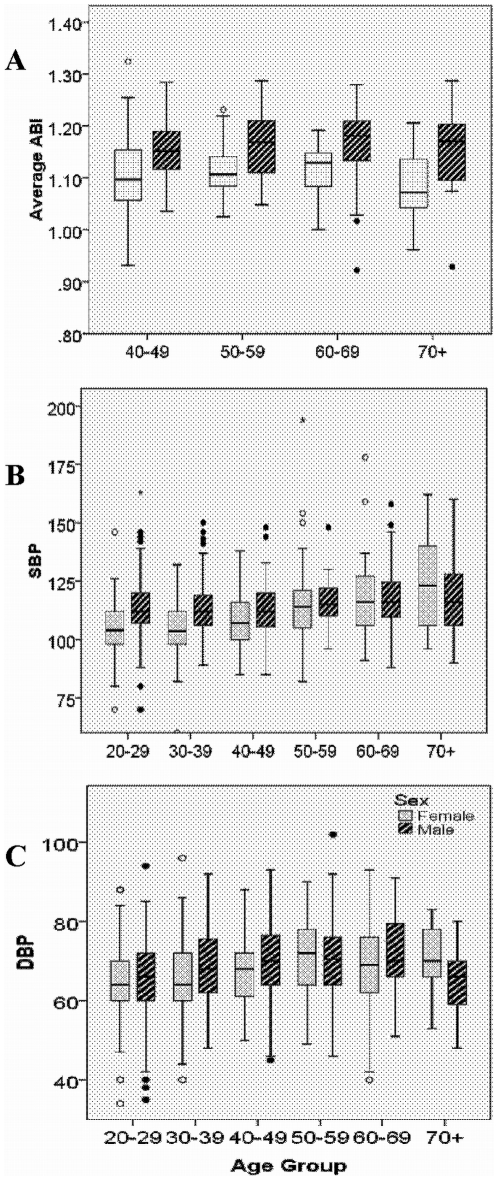
Median and interquartile values of (A) ABI (averaged across left and right sides), (B) systolic BP and (C) diastolic BP, for Tsimane by age group and sex.

PAD is absent (ABI<0.9) among all 258 Tsimane in our sample (see Methods). Mean±SD ABI is 1.10±0.07 for Tsimane women and 1.16±0.07 for men over age 40 (Fig. 1a). There is little evidence that ABI changes by age among Tsimane.

**Figure 2:**
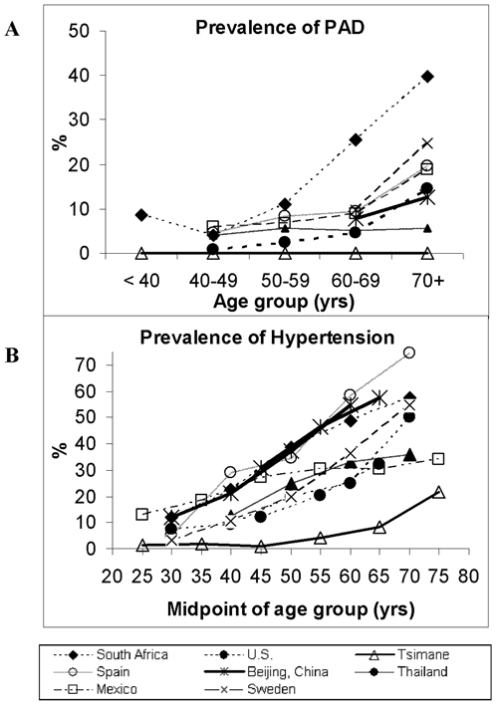
Prevalence of (A) Peripheral Arterial Disease (ABI<0.9) and (B) Hypertension (SBP≥140 and/or/ DBP>−90), among Tsimane and other populations. Data sources for ABI: urban China [67], urban Mexico [68], South Africa [28], southeast Spain [69], Sweden (Sigvant Birgitta pers comm), Thailand [70], United States [9]. Hypertension data for the same countries come from the World Health Organization Global Infobase, http://www.who.int/infobase/report.aspx. Note: x-axis represents midpoints of age intervals because of the different age intervals reported among studies (e.g. 30–39 vs. 35–44); Hypertension defined as SBP≥140 and/or DBP≥90 except for Sweden where SBP≥160 and/or DBP≥95.

The absence of PAD among Tsimane contrasts with patterns observed in national samples, especially South African blacks (Fig. 2a). PAD increases with age in every investigated population except the Tsimane, ranging from 5–25% for adults over age 70. The comparison includes people in developed and developing countries, in urban and rural settings, but none live in the relatively isolated and infected conditions of the Tsimane.

#### Blood pressure (BP)

Systolic BP was low among Tsimane young adults and climbed to moderately higher values among older adults (Fig. 1b). From age 20 to 70, the mean increases by 16% for women and 3% for men. Average diastolic BP is 9% higher for women in their 70s (65±8 mm) than for those in their 20 s, and 6% higher for older men than the average 66±10 for men in their 20 s (Table 1). Overall prevalence of hypertension among adults ≥20 years is 3.5% (SBP≥140 and/or DBP≥90), and increases with age. Hypertension peaks at 23.5% (12/51) for adults >70 years, much lower than that of the US and other countries (Fig. 2b).

**Table 1:** CVD risk factors for Tsimane population, presented as prevalence (%) of high risk, and mean absolute level, for metabolic and cardiovascular risk factors and indicators of infection and inflammation.

	Body size / lipids / lifestyle							Inflammation				Pressure		
	**Prevalence of High Risk (%) + (standard error)**													
Age group	BMI	Trig		Chol	HDL	LDL	Cig. Pack Yrs	CRP	WBC	ESR		Hyper- tension (%)		
	≥30	≥200		>240	<40	≥160	≥25	≥3	≥10800	>20 or 13		(Stage I/II)		
**40**–**49**	3.1	15.5		0.0	59.7	0.0	0.0	49.0	21.5	74.9		1.3		0.0
	(0.2)	(0.4)		(0.0)	(0.5)	(0.0)	(0.0)	(0.1)	(0.0)	(0.0)		(0.0)		(0.0)
**50**–**59**	8.3	12.0		0.0	68.1	0.0	0.0	45.1	15.0	87.6		3.7		1.9
	(0.3)	(0.3)		(0.0)	(0.5)	(0.0)	(0.0)	(0.1)	(0.0)	(0.0)		(0.0)		(0.0)
**60**–**69**	2.3	13.3		0.0	57.7	0.0	0.0	60.0	11.9	88.5		6.8		1.1
	(0.1)	(0.3)		(0.0)	(0.5)	(0.0)	(0.0)	(0.1)	(0.0)	(0.0)		(0.0)		(0.0)
**70+**	1.9	3.8		0.0	68.2	0.0	0.0	53.8	11.5	92.0		15.7		7.8
	(0.1)	(0.2)		(0.0)	(0.5)	(0.0)	(0.0)	(0.1)	(0.0)	(0.0)		(0.1)		(0.0)
	**Mean level + (standard error)**													
Age group	BMI	Trig	Chol		HDL	LDL	Cig. Pack Yrs	CRP Mean/ median	WBC		ESR	SBP	DBP	
**40**–**49**	23.9	137	144		37	80	0.4	9.9/2.7	8,968		30.1	111	68	
	(0.2)	(8)	(3)		(1)	(2)	(0.1)	(2.0)	(177)		(1.4)	(1)	(1)	
**50**–**59**	24.4	142	144		37	79	0.4	6.8/2.7	8,244		38.1	115	71	
	(0.4)	(11)	(4)		(1)	(4)	(0.1)	(1.7)	(251)		(2.3)	(1)	(1)	
**60**–**69**	23.2	116	136		37	76	0.7	7.2/4.0	8,218		38.6	118	70	
	(0.3)	(15)	(4)		(1)	(3)	(0.2)	(1.5)	(241)		(2.7)	(2)	(1)	
**70+**	22.1	121	134		35	72	0.5	15.1/3.4	8,074		47.1	121	70	
	(0.4)	(8)	(5)		(2)	(3)	(0.2)	(6.1)	(296)		(3.9)	(3)	(2)	
N =	477	203	203		172	170	463	205	480		436	472		

Note: Triglycerides and LDL are based on non-fasting samples for Tsimane (a 6+ hours fasting sample was used for US). Units for variables are the following: triglycerides, cholesterol, HDL and LDL (mg/dL), BMI (kg/m^2^), cigarette pack years (# cigarette packs smoked per day, where 1 pack-year is equal to smoking 1 pack per day for 1 year), CRP (mg/L), WBC (cells/mm^3^), ESR (mm/hr), SBP and DBP (mm Hg). For ESR, 20 is cutoff for women and 13 for men. Hypertension prevalence refers to 140≤SBP<160 and/or 90≤DBP<100 (Stage I) and SBP≥160 and/or DBP≥100.

### CVD risk factors: inflammatory markers, infectious disease and blood lipids

#### Inflammatory markers and disease risk

**Figure 3:**
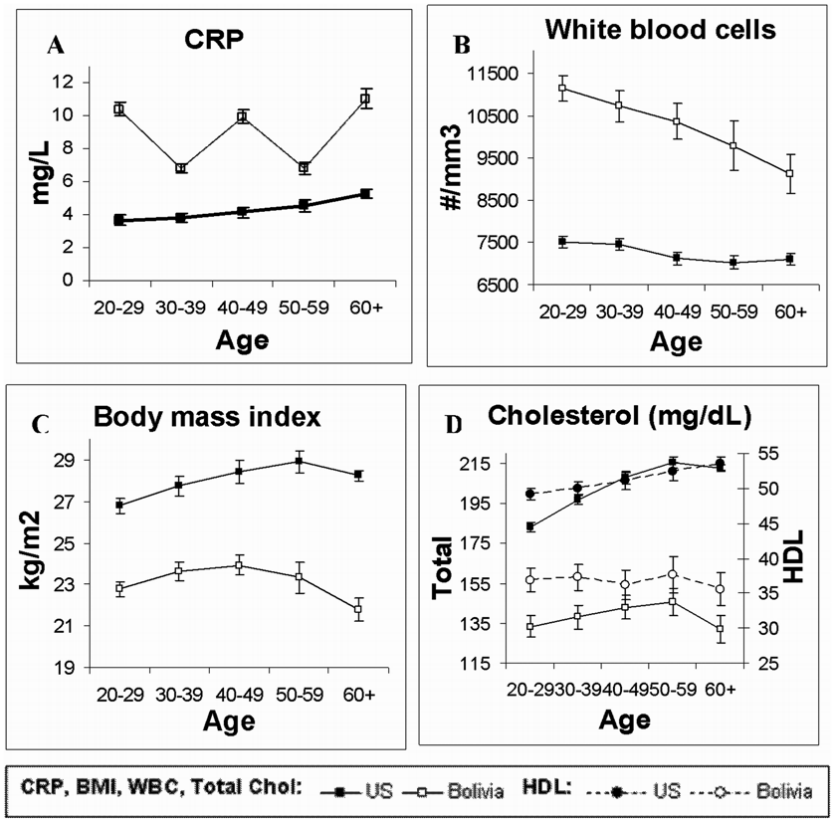
Comparison of cardiovascular disease risk factors among Tsimane and United States adults. Mean levels of (A) C-reactive protein (CRP, mg/L), (B) white blood cell (WBC) count (cells/mm^3^), (C) body mass index (BMI,kg/m^2^), (D) total and HDL cholesterol (mg/dL). Total cholesterol correlates strongly with low-density lipoprotein (LDL) among both Tsimane (r = .82, p<.0001) and US (r = .91, p<.0001), and with triglycerides (Tsimane: r = .48, p<.0001; US: r = .43, p<.0001), and so are not illustrated here. See Table 6 for further details.

Blood indicators suggest high levels of inflammation and infection among Tsimane (Fig. 3, Tables 1 and 2). For adults aged 40+, mean±SD CRP is 8.5±17.8 mg/L. About 50% of adults 40+ have CRP levels indicating CVD risk (CRP≥3.0 mg/dL) and 23% have CRP levels ≥10.0 mg/dL, which is usually indicative of acute infection [mean±SD CRP (<10.0 mg/dL) is 2.7±2.4] Tsimane white blood cell counts are elevated by US norms: with an average 9,461±2,824 units/mm^3^, 17% over 40 are elevated. The prevalence of elevated ESR is 82% (see Table 1).

**Table 2:** CVD risk factors for US population (based on NHANES 1999–2004), presented as prevalence (%) of high risk, and mean absolute level, for metabolic and cardiovascular risk factors and indicators of infection and inflammation.

	Body size / lipids / lifestyle						Inflammation		Pressure	
	**Prevalence of High Risk (%) + (standard error)**									
Age group	BMI	Trig	Chol	HDL	LDL	Cig. Pack Yrs	CRP	WBC≥	Hyper- Tension (%)	
	≥30	≥200	>240	<40	≥160	≥25	≥3	10800	(Stage I/II)	
**40**–**49**	33.9	17.8	19.1	21.4	15.2	16.3	36.3	5.7	12.7	3.0
	(1.3)	(1.6)	(1.1)	(1.0)	(1.6)	(1.1)	(1.2)	(0.5)	(1.0)	(0.5)
**50**–**59**	35.1	23.4	22.5	19.9	17.5	24.7	41.2	5.5	17.1	5.8
	(1.6)	(2.0)	(1.2)	(1.3)	(1.6)	(1.0)	(1.5)	(0.6)	(1.1)	(0.7)
**60**–**69**	38.2	26.3	22.7	19.5	16.8	32.0	45.6	4.7	23.6	11.7
	(1.0)	(1.7)	(1.0)	(1.0)	(1.4)	(1.1)	(1.5)	(0.6)	(1.2)	(1.0)
**70+**	25.7	19.0	18.9	17.0	12.4	25.3	43.3	4.3	29.3	23.7
	(1.2)	(1.5)	(0.8)	(0.9)	(1.1)	(0.8)	(1.2)	(0.4)	(1.0)	(1.3)
	**Mean level + (standard error)**									
Age group	BMI	Trig	Chol	HDL	LDL	Cig. Pack Yrs	CRP	WBC	SBP	DBP
**40**–**49**	29	159 (7.1)	208	52	125	9.6	4.1/2.0	7196	121	76
	(0.2)		(1.3)	(0.5)	(1.4)	(0.5)	(0.2)	(60.7)	(0.5)	(0.3)
**50**–**59**	29	174	214	53	128	15.1	4.6/2.4	7082	127	76
	(0.2)	(8.3)	(1.2)	(0.5)	(1.6)	(0.6)	(0.2)	(76.9)	(0.6)	(0.4)
**60**–**69**	29	162	214	53	128	20.3	5.2/2.7	7032	135	72
	(0.1)	(3.5)	(1.1)	(0.5)	(1.7)	(0.8)	(0.2)	(64.3)	(0.6)	(0.4)
**70+**	27	151	208	55	120	17.1	5.52.6	7227	144	65
	(0.1)	(2.4)	(0.9)	(0.5)	(1.1)	(0.6)	(0.2)	(57.1)	(0.8)	(0.5)
N =	8761	3871	8564	8562	3818	8641	8620	8744	8619	

Note: Triglycerides and LDL are based on 6+ hours fasting sample for US (a non-fasting sample was used for Tsimane). Units for variables are the following: triglycerides, cholesterol, HDL and LDL (mg/dL), BMI (kg/m^2^), cigarette pack years (# cigarette packs smoked per day, where 1 pack-year is equal to smoking 1 pack per day for 1 year), CRP (mg/L), WBC (cells/mm^3^), ESR (mm/hr), SBP and DBP (mm Hg). For ESR, 20 is cutoff for women and 13 for men. Hypertension prevalence refers to 140≤SBP<160 and/or 90≤DBP<100 (Stage I) and SBP≥160 and/or DBP≥100.

Tsimane clinical history is consistent with high cumulative exposure to acute infection. About 55% of those 40+ had at least one gastrointestinal illness in two prior medical exams and one third had respiratory illnesses. Two-thirds carried helminthic parasites. Infection and concomitant high inflammation are prevalent [29].

#### Blood lipids

Other CVD risk factors are low: mean±SD total cholesterol, 138±29 mg/dL and LDL, 75±22 mg/dL. However, high density lipoprotein (HDL) and triglycerides are exceptions: HDL is low at 37±9 mg/dL and trigylcerides moderately high at 130±73 mg/dL. Over half of Tsimane adults show unfavorable HDL levels by American Heart Association standards (<40 for men, <45 for women). Whereas no Tsimane show high risk levels of total cholesterol and LDL, 20% of U.S. adults have elevated levels of each blood lipid, even though many Americans use lipid-lowering medications. The prevalence of high triglycerides for most age groups in the U.S. is double that of the Tsimane. There is little indication of age increases in these measures among Tsimane. Values are similar across ages after 40 and do not show increased CVD risk at older ages in any parameter (Fig. 3, Table 1).

### Lifestyle risk factors: obesity and smoking

Obesity is 8–10 times more common in the US than among Tsimane (Tables 1 and 2). The average BMI for Tsimane adults is 24, but 29 for US adults. Among Tsimane tobacco consumption is minimal, about 0.77±1.85 pack years for men and 0.14±0.78 for women. While 72% of Tsimane men and 14% of women reported occasional use of unfiltered cigarettes or tobacco from home gardens, most Tsimane even in their sixties have smoked less than 2 pack-years. By contrast, U.S. adults aged 60+ have smoked 24 (men) and 11 (women) years.

### Summary

The main CVD risk factors greater among Tsimane than U.S. adults are markers of infection. Low HDL prevalence is also common, being about 3-fold greater among Tsimane, although the clinical significance of low HDL in energy-limited populations has not been established. Inflammatory markers (CRP, ESR, and WBC counts) were significantly higher among Tsimane (Tables 1 and 2).

#### Regressions of ABI and BP on risk factors

The links between CVD risk factors and ABI, SBP and DBP were examined in multiple regressions controlling for age, age^2^ and gender in both Tsimane and U.S. populations to explore whether risk factors show similar associations in both populations.

#### ABI

Regression results indicate that Tsimane men have higher ABI than women (Table 3), in contrast to the patterns for U.S. men and women. Obesity (BMI≥30), blood lipids, disease history, and cardiovascular indicators were not significantly associated with Tsimane ABI after controlling for age and sex (Table 3). Higher ESR predicted lower ABI (increase by 10 mm/hr associated with decrease in ABI of 0.05). Those with higher brachial diastolic BP display lower ABI. In U.S. adults, unlike the Tsimane case, higher cholesterol, CRP, WBC count, cigarette smoking and systolic pressure are all significantly associated with lower ABI, or more PAD (Table 3). Combining BMI, CRP, cholesterol and cigarette smoking in the same regression (Table 4) does not change these results. Restricting CRP analyses to cases where CRP<10 mg/L, as recommended by the American Heart Association and Centers for Disease Control [30], does not change the Tsimane results, although in the U.S. the magnitude of the effect increases five-fold (β = -0.005, p<0.001).

**Table 3:** Regression of ABI on typical risk factors and infectious disease indicators for Tsimane and US adults, 40+.

	Tsimane				United States				
Variables	beta	std err	*R^2^*	N	beta		std err	*R^2^*	N
**Baseline model:**									
Sex (ref = female)	**0.052** ***	0.009	0.145	258	**−0.041** ***		0.003	0.095	7571
Age	**0.008** *	0.004			**0.004** **		0.001		
Age^2^	**0.000** *	<.001			**0.000**		0.000		
**All analyses below control for sex, age and age^2^:**									
Total cholesterol (mg/dL)	<0.001	0.000	0.17	127	**<0.001** +		0.000	0.098	7219
Triglycerides (mg/dL)	<0.001	0.000	0.171	127	<0.001+		0.000	0.087	3343
estimated LDL (mg/dL)	0.001	0.000	0.135	110	<0.001+		0.000	0.083	3209
HDL (mg/dL)	−0.001	0.001	0.126	110	**<0.001** +		0.000	0.097	7218
Body Mass Index (kg/m^2^)	−0.001	0.001	0.141	255	<0.001+		0.000	0.093	7467
CRP (mg/L)	−0.004	0.004	0.165	129	−0.001		0.003	0.105	7261
WBC count (#/mm^3^)	0.000	0.000	0.163	232	<0.001		0.000	0.107	7353
ESR (mm/hr)	***−0.005*** +	0.003	0.173	234	**N/A**				
Cigarette pack-years	<.001	0.002	0.145	257	**−0.001**		0.000	0.120	7163
Systolic BP (mmHg)	***−0.001***	0.000	0.154	258	**−0.001**		0.000	0.125	7360
Diastolic BP (mmHg)	**−0.001** **	0.000	0.175	258	<0.001+		0.000	0.097	7360

+ p<.1.

* p<.05.

** p<.01.

*** p<.001.

**Table 4:** Multiple regression analyses of ABI, SBP and DBP among Tsimane and US.

TSIMANE	ABI (n = 126), R^2^ = 0.178		SBP (n = 260), R^2^ = 0.176		DBP (n = 260), R^2^ = 0.084	
Variable	Beta	s.e.	beta	s.e.	beta	s.e.
Intercept	0.825***	0.162	69.703***	6.864	43.965***	5.213
Male (reference = female)	**0.052** ***	0.014	**3.211** *	1.475	0.692	1.121
Age	***0.010*** +	0.005	**0.302** ***	0.052	**0.123** ***	0.039
Age^2^	***<−.001*** *	<.001	–	–	–	–
BMI	<−.001	0.002	**0.904** ***	0.236	**0.595** ***	0.179
CRP (mg/L)	−0.000	0.000	**−0.016**	0.040	**0.012**	0.030
Total cholesterol	<.001	<.001	0.035	0.025	0.026	0.019
Cigarette Pack-Years	0.003	0.010	−1.023	0.638	−0.555	0.484

UNITED STATES	ABI (n = 6746), R^2^ = 0.130		SBP (n = 7476), R^2^ = 0.214		DBP (n = 7476), R^2^ = 0.132	
Variable	Beta	s.e.	beta	s.e.	beta	s.e.
Intercept	**1.057** ***	0.039	**74.674** ***	5.773	**39.646** ***	3.951
Male (reference = female)	**−0.046** ***	0.004	1.088	0.651	**−2.414** ***	0.371
Age	**0.007** ***	0.001	**0.521** *	0.199	**1.214** ***	0.143
Age^2^	**0.000** ***	0.000	0.002	0.002	**−0.013** ***	0.001
BMI	0.000	0.000	**0.383** ***	0.048	**0.113** ***	0.030
CRP (mg/L)	**−0.001** ***	0.000	0.018	0.032	−0.022	0.026
Total cholesterol	**0.000** **	0.000	**0.038** ***	0.007	**0.027** ***	0.005
Cigarette Pack-Years	**−0.001** ***	0.000	**−0.035** **	0.012	**−0.040** ***	0.007

+ p<.1.

* p<.05.

** p<.01.

*** p<.001.

### SBP and DBP

Aside from sex, the strongest predictor of both SBP and DBP among Tsimane is BMI (standardized estimate = 0.178, 0.170, respectively). The magnitude of the effect, however, is not very large: an increase in BMI by 5 kg/m^2^ increases SBP by 4 mm and DBP by 3 mm (Table 5). High total cholesterol and triglycerides are associated with high SBP and DBP, and LDL marginally predicts higher DBP. Similar patterns for these variables are found in the U.S., with smaller BMI and triglyceride effects. In the U.S., CRP significantly associates with higher SBP, and DBP when CRP values ≥10 mg/L are eliminated (β = 0.026, p<0.001).

**Table 5:** Regression of blood pressure on typical risk factors and infectious disease indicators for Tsimane and US adults, 20+.

	Systolic BP				Diastolic BP		
	N	beta	std err	*R^2^*	beta	std err	*R^2^*
**TSIMANE: Baseline model**							
Male (reference = female)	1262	**15.500** ***	1.910	0.131	**2.920** *	1.430	0.048
Age		**0.335** ***	0.034		**0.132** ***	0.025	
Sex*age		**−0.248** ***	0.047		−0.024	0.035	
**US: Baseline model**							
Male (reference = female)	13399	**−20.600** ***	0.964	0.294	**−3.880** ***	0.811	0.015
Age		**0.373** ***	0.018		−0.016	0.012	
Sex*age		**0.393** ***	0.022		0.015	0.017	
**TSIMANE: All analyses below control for sex and age:**							
Total cholesterol (mg/dL)	383	***0.036*** +	0.019	0.135	**0.043** **	0.015	0.048
Triglycerides (mg/dL)	383	***0.014*** +	0.008	0.135	**0.012** *	0.006	0.038
estimated LDL (mg/dL)	331	0.041	0.029	0.114	***0.041*** +	0.023	0.033
HDL (mg/dL)	331	−0.026	0.071	0.108	0.064	0.056	0.026
Body Mass Index (BMI kg/m^2^)	1257	**0.782** ***	0.116	0.144	**0.534** ***	0.086	0.076
CRP (mg/L)	386	−0.005	0.028	0.126	−0.136	0.222	0.027
WBC count (#/mm^3^)	1000	0.000	0.000	0.114	**0.000** *	0.000	0.036
ESR (mm/hr)	1005	**−0.059** ***	0.018	0.123	**−0.055** ***	0.013	0.05
Mean Cigarette Pack-Years	788	**−0.789** *	0.361	0.099	***−0.481***	0.264	0.041
**UNITED STATES: All analyses below control for sex and age:**							
Total cholesterol (mg/dL)	12641	**0.027** ***	0.005	0.27	**0.041** ***	0.004	0.034
Triglycerides (mg/dL)	5758	0.003	0.002	0.259	**0.005** **	0.002	0.023
estimated LDL (mg/dL)	5566	***0.013*** +	0.007	0.263	**0.044** ***	0.006	0.034
HDL (mg/dL)	12640	−0.012	0.014	0.267	−0.012	0.012	0.016
Body Mass Index (BMI kg/m^2^)	13035	**0.417** ***	0.034	0.284	**0.270** ***	0.022	0.031
CRP (mg/dL)	12705	**0.081** **	0.022	0.263	0.006	0.016	0.015
WBC count (#/mm^3^)	12844	**0.000** ***	0.000	0.268	**0.000** ***	0.000	0.019
Mean Cigarette Pack Yrs	12430	**−0.055** ***	0.011	0.270	**−0.030** ***	0.006	0.017

+ p<.1.

* p<.05.

** p<.01.

*** p<.001.

Contrary to expectations, higher WBC and ESR associate with *lower* SBP and DBP among Tsimane, and CRP is not significant in the analyses. Restricting CRP<10 mg/L in the multiple regressions in Table 5, however, makes CRP a significant *negative* predictor of systolic pressure among Tsimane (β = −0.728, p = 0.026, n = 210), while in the U.S., CRP is a positive predictor of SBP (β = 0.579, p<0.001). Cigarette smoking among Tsimane and U.S. shows results contrary to standard prediction: higher tobacco consumption is associated with *lower* SBP and DBP (Table 5), but smoking loses significance among Tsimane in the multiple regression in Table 4.

## Discussion

Despite their high levels of inflammation, we find no evidence of advanced atherosclerosis among Tsimane adults. This is consistent with subjective clinical evaluation of arterial hardening: only 3 out of 570 individuals aged 40+ showed signs of augmented tension in the radial and humeral arteries. The presence of mild hypertension in older adults is, however, consistent with some age-related arterial stiffening. Tsimane show several clinical indicators for CVD: high blood CRP, low HDL and moderately elevated triglycerides, which are established risk factors in well-nourished populations. Tsimane diet includes salt in acculturated villages, and Tsimane consume moderate amounts of alcohol in the form of fermented manioc and maize. Nevertheless, we found little evidence for the most common risk conditions of atherosclerosis and CVD: no PAD and little hypertension. These results are consistent with reports of low CVD among traditional foraging and small-scale farming populations [22], [25]. Hypertension and CVD increase upon greater adoption of a western lifestyle [31].

Though body mass, total cholesterol, and triglycerides predict higher blood pressure among Tsimane, the magnitude of these separate effects is small, and combined do not put Tsimane at high risk. Cholesterol and triglyceride elevations of 30 mg/dL coupled with 15 kg weight gain and 20% body fat percentage increase together add <6 mm SBP and 5 mm DBP.

We propose several possible hypotheses to explain the low atherosclerosis and CVD prevalence among Tsimane, and other traditional foraging and horticultural populations living under similar conditions. The combination of low LDL and high physical exertion is a common feature in many of these populations. The Tsimane diet contains wild game and fish, is low in saturated fat, and high in potassium (K). Plantains provide ∼1,500 mg K/day. Low BMI and LDL, and sparse tobacco consumption may be protective factors trumping the risk factors of atherosclerosis and CVD. Oxidized LDL is implicated in inflammatory cascades leading to endothelial dysfunction, plaque maturation and rupture; therefore some argue that atherosclerosis and CVD are avoidable when LDL is maintained <70 mg/dL [15]. The Tsimane LDL profile is favorable though only 34% of Tsimane adults age 40+ have LDL below 70. Mean BP and ABI, however, do not differ among those above this LDL cutoff. With low HDL and moderate triglyceride levels, Tsimane have a healthy but “normal” mean total cholesterol to HDL ratio of 4.0 and an LDL to HDL ratio of 2.3 (for adults age 40+).

The physically demanding Tsimane lifestyle may be central for maintaining healthy metabolism and favorable body mass, blood lipids and cardiorespiratory health. Subsistence hunting, fishing and slash and burn farming require extensive daily physical activity, consistent with high VO_2_max values found using a variation of the Harvard Step Test on a subsample. Using equations of total energy expenditure with body weights and physical activity levels (PALs) for Tsimane and relatively sedentary U.S. adults [32], we estimate that Tsimane men and women age 40–49 expend 850 and 450 kcals/day more, respectively, in physical activity than U.S. adults. To achieve mean Tsimane adult BMI, U.S. adults would need to expend 200–300 kcals/day above current levels. Two-thirds of U.S. adults age 18+ never engage in vigorous leisure-time physical activities lasting 10 minutes or more per week, and only 25% engaged in such activity 3+ times per week [33]. Only 15% of U.S. adults engage in moderate-to-vigorous physical activity for 30 minutes or more per day as recommended by the CDC/ACSM and stated in the *Healthy People 2010* objective. A recent study using an accelerometer rather than self-report showed that <5% of U.S. adults met this criterion [34]; in contrast, Tsimane adults are physically active most days of their lives.

These results are consistent with evidence for the cardioprotective value of exercise. Exercise reduces oxidative load in muscle, levels of inflammatory cytokines, SBP, macrophage-rich fat and improves insulin sensitivity [10]. Exercise associates with a favorable CVD risk profile independently of leanness [35]. Individuals with high cardio-fitness based on resting and maximal heart rate and VO_2_max show the lowest heart disease risk [36], [37]. Even Sumo wrestlers, despite intentional obesity (BMI>35; >25% body fat), have normal blood lipid levels during training periods, but then suffer from premature morbidity and mortality after retiring in their mid−30's [38]. Industrialization and automation of manual labor have diminished physical activity in most occupations such that much physical activity in the 20^th^ century and beyond comes from sports and leisure. Even prior to entering the workforce, physical activity is extremely low among US adolescents [34].

Several alternative explanations may also be responsible for the low CVD risk profile of Tsimane, and merit future investigation. Tsimane and other Amerindians should be comprehensively investigated for distinct inflammation profiles due to genetic variability in loci affecting expression of CRP [39], apolipoprotein-E [40], mannose-binding lectin (MBL) [41], NR4A nuclear receptor family [42], interleukine-6 (IL-6) [43], IL-1 [44], and toll-like receptor 4 [45]. These reflect the extent to which Tsimane exhibit pro- or anti-inflammatory preconditions. Amerindians also show distinct human leukocyte antigen (HLA) expression at various MHC loci compared with other populations that show evidence of overdominant selection [46]. Although HLA-DR expression in macrophages and T-cells has been linked to plaque eruption and erosion [47], it is an open question whether allelic variation is of clinical significance. Other genetic contributions involve the processes by which inflammation and arterial damage result in acute coronary events. Plaque stability, disruption, erosion and thrombogenicity may be impacted by genes affecting interferon-α, collagen content and smooth muscle density in the fibrous cap [48], inflammatory infiltration of the cap, lipid composition of the atheromatous core and extracellular matrix (e.g. soft cholesterol esters vs. hard crystalline cholesterol), and both mechanical and hemodynamic forces that shear apart plaques. Genetic differences affecting metalloproteinase expression (e.g. collagenases, gelatinases, stromelysins and matrilysin) may also be important, as these have been described as degrading all components of the extracellular matrix and can therefore also impact plaque disruption [49]. Genetics and diet (e.g. flavonol-rich citrus fruits) may also influence platelet aggregation, coagulation and fibrinolysis, which affects blood flow and thrombus formation at the site of disrupted plaques [50]. Genes affecting monocyte recruitment [e.g. CD14 receptor polymorphisms] [51], lipid transport [e.g., cholesteryl ester transport protein (CETP)] [52], lipid oxidation, and modulation of the inflammatory response to oxidized lipids may also help explain differences in susceptibility of populations to developing atherosclerosis [53].

One preliminary attempt to assess the implications of genetic differences between Tsimane and other populations is to consider atherosclerosis and CVD risk among Amerindians in the U.S. A study among 13 North Amerindian groups revealed a low rate of PAD (5.3%) among adults aged 45–74, little difference in PAD prevalence among the groups, and significant predictive effects of LDL, BMI, cigarette pack-years and fibrinogen [54]. Thus, while PAD is on the low end among North Amerindians, PAD is associated with the same risk factors as those in other populations. Most importantly, CRP levels among North Amerindians are higher than in other U.S. populations (median 3.2 mg/L even after removing the 16% who had CRP>10 mg/L), and predict incident CVD [55]. Despite similar PAD rates and lower LDL levels than national averages, a longitudinal cohort study of CVD among North American Indians has shown that North Amerindians have higher rates of CVD than other U.S. populations, and that standard risk factors (e.g. hypertension, LDL, HDL, BMI) are predictive of CVD [56]. Their high rates of CRP and CVD are likely due to the high prevalence of diabetes, renal disease and obesity; hyperglycemia impedes endothelial function and produces glycation end products that support myocardial dysfunction [57].

Another unexplored potential explanation highlights the hypothesized cardioprotective effects of chronic helminthic infection. Polarized Th-2 immune activation associated with helminthic infection modifies cytokine profiles, whereby anti-inflammatory IL-4, IL-10 and IL-13 protect vessel walls from oxidized LDL-induced monocyte injury in the endothelium, and downregulate fibrinogen synthesis [58]. Th-2 activation may also modulate responses to heat shock proteins, *Chlamydia pneumonia*, and cytomegalovirus, each of which has been tentatively linked to atherosclerosis [59]. Finally, helminthes may modulate host lipid metabolism, and may be responsible for lower blood cholesterol levels in parasitized humans [60]. Indeed, total and HDL cholesterol varied inversely with several infectious markers such as sedimentation rate, IgE and eosinophil count among Tsimane adults [61].

### Conclusion

Though our characterization of arterial disease is provisional pending ultrasonographic studies, our study provides evidence that chronic low-grade inflammation in the absence of several other risk factors is not a determinant of CVD in a subsistence population. Inflammation and infection may not accelerate arterial degeneration in the context of restricted caloric intake, parasitism, and daily physical activity that maintains low BMI. We observed low levels of atherosclerosis and associated CVD among Tsimane, suggesting that these conditions may have been rare throughout pre-industrial human history. However, as indigenous populations like the Tsimane rapidly acculturate to western lifestyles, rates of CVD among older adults may rise considerably. Transitioning populations exhibiting western lifestyles but relatively high pathogen load are likely to suffer the double burden of chronic and infectious disease morbidity and mortality [62], [63].

## Materials and Methods

### Ethics Statement

Informed consent was obtained for all protocols at three levels: 1) *Gran Consejo Tsimane*, the local Tsimane government organization that represents Tsimane interests and oversees all projects, 2) community officials and participants in village meetings, and 3) individual consent during medical visits and before each procedure. After explanation of a formal protocol by bilingual Tsimane assistants, consent forms were signed for literate participants, and verbal approval with fingerprint signature given for non-literate participants. Our consent procedures have been approved by the Institutional Review Boards at the Univ. of New Mexico, Univ. of California-Santa Barbara and Univ. of S. California.

### Tsimane

Data were collected during annual medical exams of the Tsimane Health and Life History Project co-directed by MG and HK (www.unm.edu/~tsimane). Table 6 provides summary information on the sample population. Volunteers aged 40+ in 15 villages were sampled for ABI, blood pressure and anthropometry (2006 census population: 2,324 individuals; 350 were 40+, final sample *n* = 259, 133 or 52% male; 05–12/2007). Blood pressure was recorded from a larger sample of volunteers aged 20+ in 25 villages (eligible population 3,482; 1,298 are 20+; *n* = 1,262, 50% male; 11/2005–12/2007).

**Table 6:** Sample characteristics for key study variables in analyses of ankle-brachial index (ABI) and blood pressure (BP).

Variable	Tsimane				United States (NHANES)			
	40+ ABI sample		20+ BP sample		40+ ABI sample		20+ BP sample	
	Mean	N	Mean	N	Mean	N	Mean	N
Sex (%)	51.6	258	50.8	809	51.7	7571	51.6	13399
Age (yrs)	53.1	258	37.6	1604	56.2	7571	46.1	13399
Height (cm)	156.8	257	156.1	1280	168.7	7479	169.0	13137
Weight (kg)	62.2	257	58.8	1280	80.8	7530	80.2	13149
BMI (kg/m2)	25.2	256	23.7	1266	28.3	7467	28.0	13035
Systolic BP	113.2	258	110.6	1262	128.7	7360	123.6	13399
Diastolic BP	71.1	258	67.4	1263	73.4	7360	71.7	13399
ABI	1.13	258	1.13	242	1.13	7571	1.13	7360
CRP (mg/L)	8.47	129	9.35	430	4.4	7260	4.2	12705
median	2.33		2.64		2.30		2.10	
Total Chol (mg/dL)	146.5	127	137.6	427	210.7	7219	202.8	12641
HDL (mg/dL)	38.1	110	36.7	369	53.2	7218	52.2	12640
LDL (mg/dL)	80.6	108	74.8	366	126.1	3209	121.2	5566
Triglyceride (mg/dL)	134.9	127	129.8	427	162.2	3343	148.2	5758
Cigarette pack-yrs	0.4	257	0.4	800	14.5	7162	10.3	12429

In-field blood analysis of nonfasting venous samples provided estimates of WBC and ESR. Blood measures exist for 234 ABI and 1,000 BP samples. CRP, total cholesterol, HDL and triglycerides were analyzed separately on a subset of serum samples by TriCore Laboratories (Albuquerque, NM). ABI exists for >110 of the 203 (age 40+), and blood pressure for 383 of the 427 (age 20+) samples with blood lipids, CRP and antibodies. Non-fasting LDL is estimated using the Friedewald formula (total cholesterol – HDL – triglycerides/5). CRP, ESR, and WBC are biomarkers of inflammation and infection.

Physicians measured ABI after training by HK and MG according to standard protocol by the American Heart Association. The patient lies supine with feet uncovered while brachial and ankle systolic pressure measurements are made using a SummitDoppler L150 ultrasound machine. Systolic pressure is first measured in the posterior and anterior tibial arteries and the higher of these is selected as the ankle measurement for each foot. The cuffs are inflated on the ankle to roughly 30 mm Hg above the systolic pressure, then followed by a slow release until the first audible sound of systolic pressure is heard. Systolic pressure is then measured in each arm and the highest is chosen as the brachial pressure. The ratio of the left and right ankle pressures to the brachial pressure is the left and right ABI. ABI <0.9 indicates PAD. ABI between 0.5 and 0.9 corresponds to intermittent claudication in the lower limbs, whereas values <0.5 are associated with more severe symptoms such as resting pain, severe occlusion and critical ischemia. ABI values >1.3 suggest calcification of arterial walls and noncompressible vessels, and are therefore also symptomatic of severe PAD.

Systolic (SBP) and diastolic (DBP) pressure were measured during each visit with a Welch Allyn Tycos Aneroid 5090 sphygmomanometer and Littman stethoscope. The systolic brachial pressure using the Doppler highly correlates with aneuroidal SBP (r = 0.896, p<0.0001). Cumulative experience smoking cigarettes was measured in cigarette pack years based on interviews. One pack year is equal to a pack of cigarettes smoked per day for one year. Bolivian physicians using bilingual Tsimane assistants diagnosed illnesses and trauma presented by patients on annual visits since 09/2002. Diagnoses from the International Classification of Disease (ICD-10) are grouped into gastrointestinal, respiratory, and other infections, over the two most recent exams 10/2005–12/2007.

### Age estimation

Age estimates are derived from demographic interviews conducted with all individuals aged 18+ (n = 1,098) and from missionary records. Years of birth were assigned based on methodologies employed by researchers among the !Kung [64], Ache [65] and Hadza [66]. These include using known ages from written records, relative age lists, dated events, photo comparisons of people with known ages and cross-checking of information from independent interviews of kin. For example, Catholic missionaries have recorded the dates of 1,110 births among the Tsimane since 1952, many of the deaths occurring during the same period, and age estimates for an additional 120 individuals who were baptized as small children or as young adults during the early 1950's. In constructing relative age lists, multiple informants were used for each five year age grouping of individuals and inconsistencies were investigated and resolved. The photo comparison method used a sample of seventy photos of individuals with known ages. For older individuals, fifty photos of men and women from ages 50 through 75 were used. Each of these methods provides a roughly independent age estimate. When all estimates yield a date of birth within a 3-year range, the average was used unless one or two estimates were judged to be superior to the others. Further details are given in [1].

### United States

Tsimane health status was compared with NHANES data for the years 1999 to 2004 (N = 7,571 for adults age 40+, N = 14,213 for adults age 20+). The NHANES monitors the health and nutrition of a representative sample of the American noninstitutionalized population. Methods have been widely published and so are only briefly summarized here.

Lipid indicators include total, LDL and HDL cholesterol and triglycerides. Triglycerides and LDL were measured for approximately half of the sample that fasted for at least 6 hours. Total and HDL cholesterol and triglyceries were assayed using the Hitachi 704/717/912 Analyzer, Roche Diagnostics. Fasting LDL cholesterol was calculated using the Friedewald equation. CRP was determined by the latex-enhanced Behring Nephelometer. White blood cell differentials were determined using the Beckman Coulter® MAXM.

ABI was measured among the 40+ sample (N = 7,571) by trained technicians. People who had amputations, excessive obesity, or other conditions that inhibited examination were excluded. The procedure was the same as described for the Tsimane. Systolic and diastolic blood pressure was measured by physicians using a stethoscope and sphygmomanometer (Baumanometer® with a Calibrated® V-Lok® cuff, Latex Inflation Bulb, and an Air-Flo® Control Valve). SBP and DBP values are the average of three individual readings.

### Analysis

Multiple linear regression (PROC REG in SAS 9.1) was used for continuous values of ABI and blood pressure, as a function of demographic variables, risk factors and disease markers. Each risk factor is included separately in a baseline model that controls for sex, age and age^2^ for ABI, and sex, age and sex*age for BP. These results are reported in Tables 3 and 5. Table 4 reports multiple regressions of ABI and BP that include BMI, CRP, cholesterol and cigarette smoking.

Post-hoc power analysis for multiple regression assuming a 0.05 alpha level was performed to assess whether the absence of significant effects in the Tsimane analyses was due to small sample size. Given the observed *R^2^*, number of predictors and sample size, most regressions show power above 92%. Even considering the analysis of HDL on ABI (Table 3), where the sample size and *R^2^* were lowest, power was still 89.6%. Only in the regressions of diastolic BP (Table 5), where *R^2^*<0.08, did power reach as low as 70% (for HDL). The only analyses of Tsimane DBP that did not reveal statistically significant effects, however, also were not significant in the U.S. analyses, where power was 100%.
